# An Unusual Prepatellar Bursa Swelling: Patellar Button Dissociation and Migration

**DOI:** 10.1155/2016/8208271

**Published:** 2016-01-17

**Authors:** Thomas Hester, Farid Moftah

**Affiliations:** ^1^Guys and St Thomas' NHS trust, Department of Orthopaedics, Westminster Bridge Road, London SE1 7EH, UK; ^2^Darent Valley Hospital, Darenth Wood Road, Dartford, Kent DA2 8DA, UK

## Abstract

Implant loosening is not a new phenomenon, nor is implant migration; however they are rarely seen after knee arthroplasty surgery. Complications with patellar buttons have been reported before with peg failure, loosening, and patella fracture; however extra-articular migration is extremely rare. We report an unusual case of patellar button migration 11 years after total knee arthroplasty to the prepatellar bursa.

## 1. Introduction

Total knee arthroplasty is an excellent and reliable operation, but opinion regarding the need for patella resurfacing remains controversial amongst knee arthroplasty surgeons. Patella resurfacing has been shown to reduce the prevalence of anterior knee pain following total knee arthroplasty with improved patient satisfaction scores. Opponents of patella resurfacing will argue the unnecessary added complications of patella maltracking, subluxation, dislocation, fracture, or implant loosening that have been associated. However, loosening followed by implant dissociation and extra-articular migration is extremely uncommon [1, 2]. There has been a case report of a recurrent patella component loosening with subsequent extra-articular migration [3]. We report a similar case of extra-articular implant migration, but it is unique because this was the first episode of aseptic loosening and migration through a lateral capsular defect in a patient that was aesthetically unfit for surgery.

## 2. Case Report

**Figure 1:**
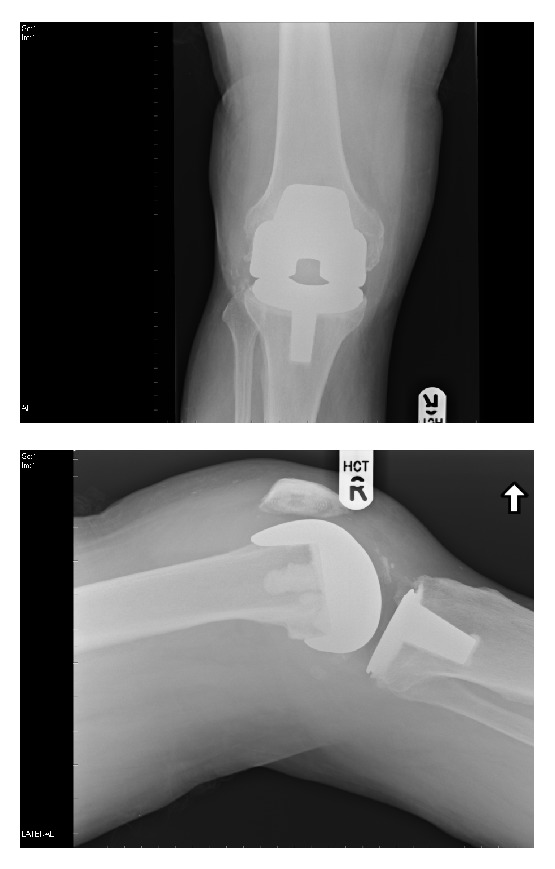
Initial operative AP and lateral radiographs of the right knee.

We report a 76-year-old gentleman who had a right Anatomic Graduated Component (AGC) (Biomet Ltd, UK) cemented total knee arthroplasty 11 years previously (Figure 1) at another centre with no immediate postoperative complication reported, who presented with a prominent mobile mass over the right knee. The knee was otherwise functioning well with minimal discomfort and he had only noticed the mass for the previous 2 weeks when he felt it arise from inside the knee after a twisting motion whilst getting up. Medical history was significant for atrial fibrillation, obstructive cardiomyopathy with poor left ventricular function, previous myocardial infarction, hypertension, and a body mass index of 40. Despite these comorbidities he was able to mobilise himself indoors holding onto furniture and outside with a stick for approximately 200 yards.

**Figure 2:**
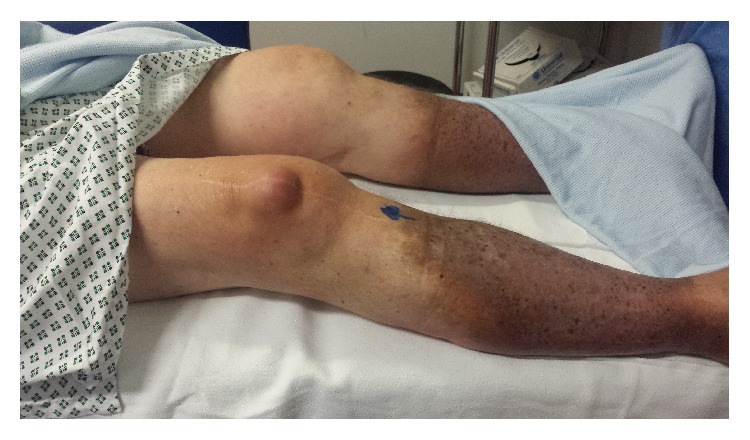
Clinical photo of the right knee showing a swelling anterior to the patella.

**Figure 3:**
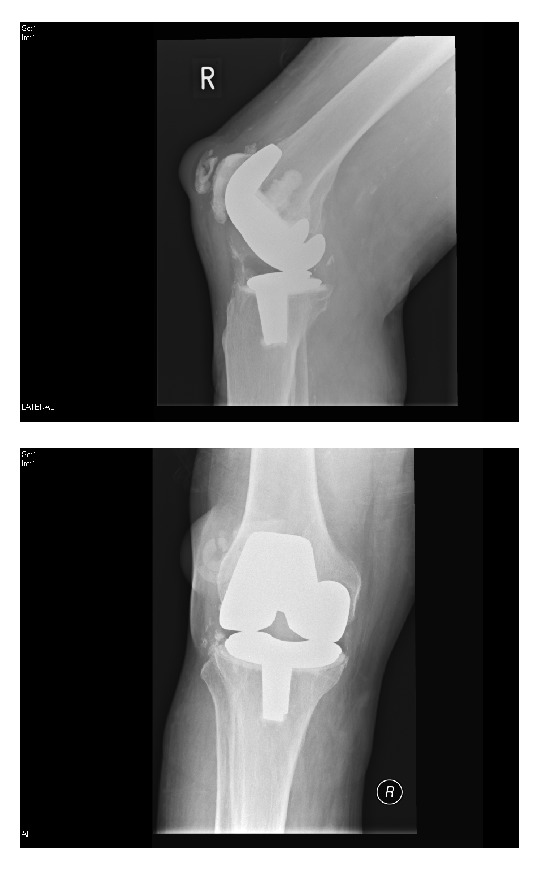
Postoperative AP and lateral radiographs of the right knee showing that the patellar button has migrated anteriorly to the patella.

**Figure 4:**
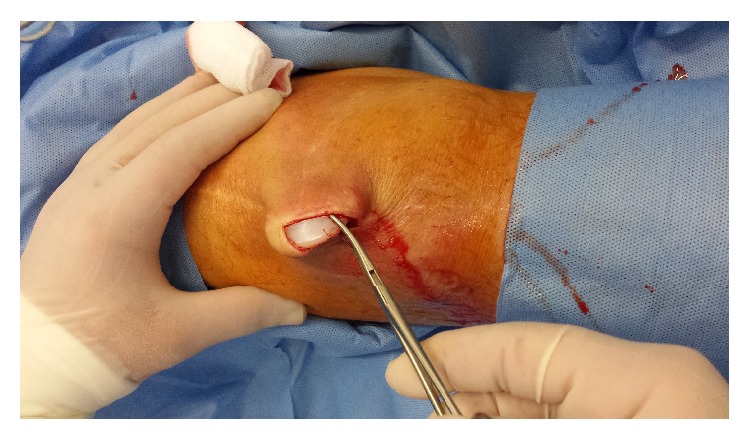
Intraoperative photo demonstrating the patellar button superficial to the patella.

Examination demonstrated a well-healed midline scar and no effusion but a prominent swelling anterior to the patella. He had a range of motion from 5 to 100 degrees with minimal discomfort except at the end of flexion due to tightness over the patella caused by the mass with blanching of the skin (Figure 2). The swelling was mobile and smooth and after further investigation found to be the patellar button which was aseptically loose (Figure 3). Due to his significant medical comorbidities, the decision was made along with the patient, relatives, and anaesthetist that the button would be removed under local anaesthetic and a formal revision would not be undertaken at this point (Figure 4). Postoperatively he made an uneventful recovery and was back to his premorbid mobility at the 6-month review.

Inspection of the implant found that the single peg was intact and no fracture at the stem-button interface was seen. The cement mantle was shown to have a good imprint of the prepared patella surface indicating good preparation and pressurisation at the time of implantation.

## 3. Discussion

Patellar button dissociation is an uncommon phenomenon, even more so when the implant migrates extra-articularly through the retinaculum and rests above the patella at the level of the prepatellar bursa [3, 4]. Reported patellar button dissociation has occurred at the level of the peg button interface and was suspected before implant retrieval on this occasion [2, 5]. The nature and path of the migration are also unusual, with the patellar button lying within the prepatellar space. Unfortunately the original operation note was not available for review, but at the time of retrieval, a defect was found in the lateral retinaculum rather than the medial side where a defect in a medial parapatellar approach repair could explain the path of the implant as described in another case [6]. The patient described a varus deformity preoperatively and so a lateral parapatellar approach is unlikely. We hypothesised that an extensive lateral release may have provided the opening for the implant to migrate through the soft tissues.

Along with other reported complications of patella resurfacing during total knee arthroplasty, this unusual complication and also the risks of future component revision should also be borne in mind when selectively resurfacing the patella. In this scenario, with a high-risk patient, a significant challenge is faced by the surgeon to balance the patients fitness and the ideal of revising the implant. There is one previous case reported of a conservatively managed extra-articular patellar component by Helito et al. [7]. In that case the implant had already partially extruded through the skin; the implant was simply removed and the wound closed. We believe this is the first report of such a case with a patellar button found anterior to the patella and successfully managed this way. An argument may have been made for leaving the implant in situ; however with the blanching of the skin and the natural history of the extruded patellar button as described by Helito et al., where the implant came through the skin, it would seem advisable to remove the implant [7]. We hope this demonstrates that despite there being an accepted first-line method of revision resurfacing, often surgeons have to be more pragmatic with their approach [5].
